# A new approach to construct pathway connected networks and its application in dose responsive gene expression profiles of rat liver regulated by 2,4DNT

**DOI:** 10.1186/1471-2164-11-S3-S4

**Published:** 2010-12-01

**Authors:** Sudhir Chowbina, Youping Deng, Junmei Ai, Xiaogang Wu, Xin Guan, Mitchell S Wilbanks, Barbara Lynn Escalon, Sharon A Meyer, Edward J Perkins, Jake Y Chen

**Affiliations:** 1 Indiana University School of Informatics, Indianapolis, IN 46202, USA; 2 Rush University Cancer Center, Rush University Medical Center, Chicago, IL 60612, USA; 3 School of Computing, University of Southern Mississippi, Hattiesburg, MS 39406, USA; 4 SpecProc Inc., Vicksburg, MS 39180, USA; 5 US Army Engineer Research and Development Center, 3909 Halls Ferry Road, Vicksburg, MS 39180, USA; 6 Department of Toxicology, University of Louisiana at Monroe, Monroe, LA 70804, USA; 7 Department of Computer and Information Science, Purdue School of Science, Indianapolis, IN 46202, USA; 8 Indiana Center for Systems Biology and Personalized Medicine, Indianapolis, IN 46202, USA

## Abstract

**Abstract:**

**Background:**

Military and industrial activities have lead to reported release of 2,4-dinitrotoluene (2,4DNT) into soil, groundwater or surface water. It has been reported that 2,4DNT can induce toxic effects on humans and other organisms. However the mechanism of 2,4DNT induced toxicity is still unclear. Although a series of methods for gene network construction have been developed, few instances of applying such technology to generate pathway connected networks have been reported.

**Results:**

Microarray analyses were conducted using liver tissue of rats collected 24h after exposure to a single oral gavage with one of five concentrations of 2,4DNT. We observed a strong dose response of differentially expressed genes after 2,4DNT treatment. The most affected pathways included: long term depression, breast cancer regulation by stathmin1, WNT Signaling; and PI3K signaling pathways. In addition, we propose a new approach to construct pathway connected networks regulated by 2,4DNT. We also observed clear dose response pathway networks regulated by 2,4DNT.

**Conclusions:**

We developed a new method for constructing pathway connected networks. This new method was successfully applied to microarray data from liver tissue of 2,4DNT exposed animals and resulted in the identification of unique dose responsive biomarkers in regards to affected pathways.

## Background

2,4-Dinitrotoluene (2,4DNT), which has been found to contaminate both soil, surface water and groundwater, is used in production of polyurethane foam, propellants and as a plasticizer in explosives [1]. 2,4DNT has been found to be toxic to reproductive organs in rats [2] and cause genetic toxicity in munitions facility workers and copper miners using explosives [3,4]. DNTs including 2,4DNT are listed as a priority pollutant by the U.S. Environmental Protection Agency [3]. It is therefore important to develop methods to biomonitor people and animals exposed to nitrotoluenes to prevent such potential harmful effects.

Toxicogenomics using microarray technology has recently been applied to 2,4DNT and other military compounds to understand its molecular mechanisms[5-10]. While little information exists on the molecular pathways affected by 2,4DNT in mammals, mechanisms of 2,4DNT toxicity have been explored in the ecotoxicological fish model *Pimephales promulas.* Expression analysis of the effects of 2,4DNT on *P. promulas* indentified molecular pathways involving oxidation of hemoglobin and alteration of lipid metabolism via peroxisome proliferative activator receptor alpha signaling as being involved in 2,4DNT induced toxicity [8,11].

In toxicogenomics, molecular expression pattern changes of cells occur as a result of exposure to toxicants and give insight into how toxicants act and cause disease. Nonetheless, many of these patterns are complex, interconnected, and reflect a dynamic process evolving from exposure to disease. Therefore, it is necessary to develop a systems approach to studying toxicogenomics by integrating ‘omics' measurements with public domain knowledge and interpreting them with a computational approach that encodes such a comprehensive biological context [12]. Examples of such knowledge integration applied to systems biology include gene ontology analysis [13], protein-protein interaction network analysis [14], biological pathway analysis [15], and visualization of complex biological networks [16]. However, few successful examples have been demonstrated in toxicogenomics.

In this work, we aim to study systems-level functional toxicogenomic changes induced by 2,4DNT exposure, using rat liver as an experimental model. We employ a new comprehensive pathway database resource, the Human Pathway Database (HPD) (Chowbina, Wu et al. 2009), which contains a comprehensive collection of human annotated and predicted pathway data. We mapped significantly-changed expression patterns of rat genes to human genes, and used HPD pathways to help us interpret complex interconnected gene expression patterns. We compared different dose effects of 2,4-DNT and the broad pathway-level changes the compound induced. To connect changes brought into different pathways, we further constructed a pathway- connecting-network (PCN) specific to differentially expressed genes induced by 2,4-DNT. This systems-level toxicogenomics study enables us to identify four major pathways implicated with 2,4-DNT induced cellular toxicity including long-term depression, breast cancer regulation by stathmin1, retinol metabolism, and WNT signaling. Genes significantly involved in these processes include PPP2R2B, PLCB1, CACNA1D, PTPRD, PTPRG, and RDH16.

## Results

### Dose responsive differentially expressed genes in rat liver after 2,4DNT exposure

Female Sprague-Dawley rats were dosed with vehicle control (5% DMSO in corn oil) or 2,4DNT at 4.98, 49.8, 99.5, or 199 mg/kg. After 24 hrs, rats were sacrificed, livers harvested and gene expression examined in liver using whole genome microarrays. Compared to controls, samples treated with 2,4DNT had many differentially expressed genes (DEGs) (Table 1). Genes were selected for further analysis only if they passed the following criteria: false discovery rate < 5%, fold change > 2 and q-value < 0.05. The DEGs in the fourth column are those genes which have a higher expression amount than control samples. Notably, a clear dose response relationship was observed between increasing dose and increasing numbers of DEGs. However, no significant effect on transcription was observed at the lowest dose of 2,4DNT after 24 hrs.

**Table 1:** Statistical analysis of microarray results

Dose (mg/kg)	FDR (< 5%)	Median no. of false positives	No. of significant differentially expressed genes relative to controls (q-value < 0.05)
4.98	0	0	0
49.8	4	4.72	117
99.5	4.55	59.43	1260
199	4.66	189.59	4148

### Pathway analysis using genes differentially expressed at each concentration of 2,4DNT

**Figure 1:**
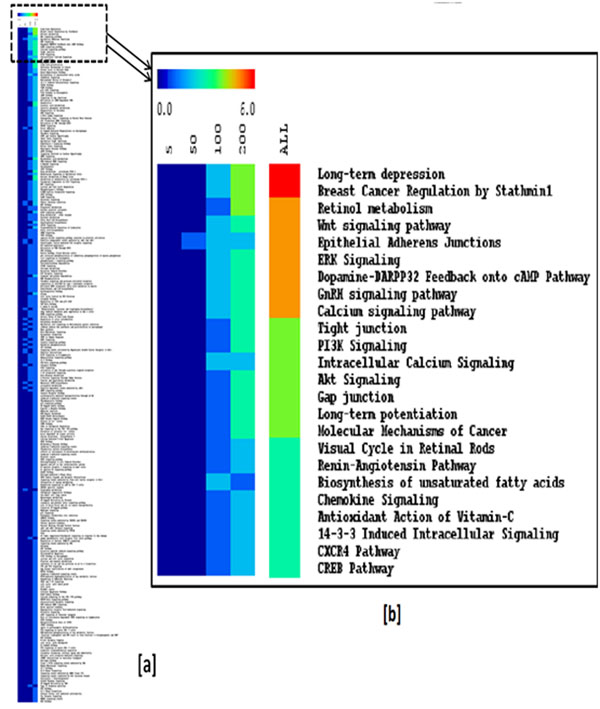
**Protein frequency count matrix (PPFCM) generated using differentially expressed genes at three doses of 2,4DNT treatment** [a] 266 pathways in the pathway. The vertical axis contains pathways from the Human Pathway Database (ref) pathways and doses, rounded to nearest whole number, on the horizontal axis. Each cell of PPFCM corresponds to the count of proteins at a particular dose for a given pathway. [b] Snapshot of top 25 pathways. The pathways are ranked in a descending manner based on the sum of DEGs at ‘ALL’ doses.

We used DEG data from 49.8, 99.5, and 199 mg/kg exposures to build dose-specific protein- protein association matrix and dose-response networks in addition to using the Human Pathway Database (HPD) to identify potential pathways affected by 2,4DNT [17]. The driving motivation is that knowledge of these pathways/protein-protein interaction networks will help clarify and interpret physiological responses to 2,4DNT. A novel algorithm was devised to generate a pathway-protein frequency count matrix (PPFCM) using DEGs from each of the three doses of 2,4DNT (Figure 1). A PPFCM contains pathways on the vertical axis and doses on the horizontal axis. Each cell of PPFCM corresponds to the count of proteins (mapped from) at a particular dose for a given pathway. The pathways affected by 2,4DNT were ranked in a descending manner based on the sum of DEGs at ‘ALL’ doses. The PPFCM indicated that signal transduction, neurological, and metabolic pathways were impacted including long-term depression, breast cancer regulation by stathmin1, WNT signaling, and PI3K signaling.

### Dose responsive pathway connected networks

**Figure 2:**
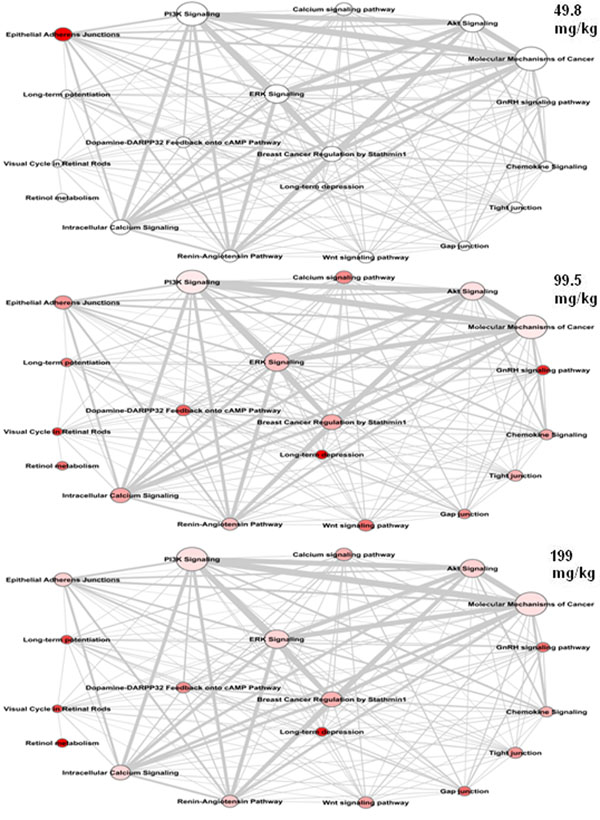
**Pathway merging network of top 20 enriched pathways affected by 2,4DNT treatment at each of the three doses.** The network was derived from differentially expressed genes (DEGs) across all doses of 2,4DNT. The size of each node is proportional to the number of proteins in a pathway. The color of each node reflects the intensity of the DEGs in each pathway with darker shades of red indicating greater numbers of DEGs. The edge label width is proportional to the number of molecular entities shared by the connected pathways.

The top 20 pathways identified by PPFCM analysis and derived from HPD were used to build a pathway merging network with pathway similarity scores for each dose (Figure 2). To merge the top 20 pathways, we used the concept of pathway similarity and applied a minimal pathway similarity threshold [*S_i,j_* ≥ 0.2, and |*P_i_* ∩*P_j_*| > 1], *i*=1...*N*, *j*=1...*N*. At this threshold, two pathways are considered connected if at least 20% minimal molecular entities are shared and there is no less than 1 shared entity. Only pathway pairs with a similarity score and overlap above the threshold [*S_i,j_* ≥ 0.2, AND |*P_i_* ∩ *P_j_*| >1] are shown.

**Figure 3:**
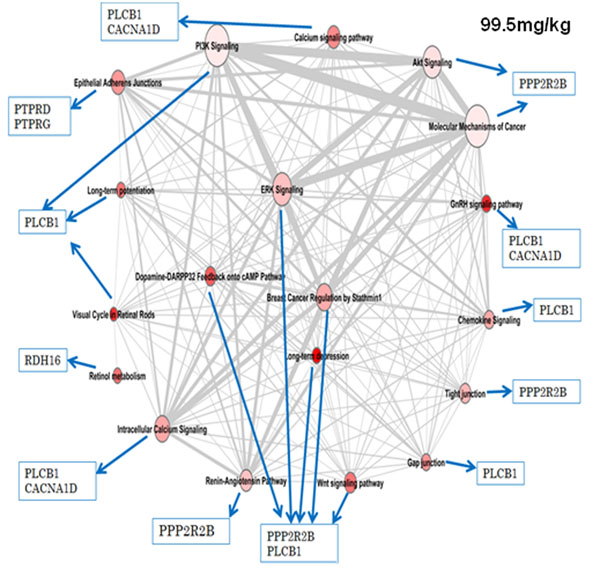
**Location of 2,4DNT impacted genes in the pathway association network.** Select genes that are differentially expressed in 99.5 mg/kg 2,4DNT exposures are indicated by blue arrows. PPP2R2B = Serine/threonine-protein phosphatase 2A 55 kDa regulatory subunit B beta isoform. PLCB1 = 1-phosphatidylinositol-4,5-bisphosphate phosphodiesterase beta-1. CACNA1D = Voltage-dependent L-type calcium channel subunit alpha-1D. PTPRD = Receptor- type tyrosine-protein phosphatase delta; PTPRG = Receptor-type tyrosine-protein phosphatase gamma. RDH16 = Retinol dehydrogenase 16.

Three data sources, were used to generate a comprehensive perspective of pathways which might be affected by 2,4DNT. The network corresponding to dose 99.5 mg/kg can be further represented at gene level as shown in Figure 3. The genes and full names are: (a) PPP2R2B - Serine/threonine-protein phosphatase 2A 55 kDa regulatory subunit B beta isoform; (b) PLCB1 - 1-phosphatidylinositol-4,5-bisphosphate phosphodiesterase beta-1; (c) CACNA1D -Voltage- dependent L-type calcium channel subunit alpha-1D; (d) PTPRD - Receptor-type tyrosine- protein phosphatase delta; (e) PTPRG - Receptor-type tyrosine-protein phosphatase gamma; and (f) RDH16 - Retinol dehydrogenase 16.

## Discussion

In this manuscript, we identified biological pathways and processes that may be biomarkers of 2,4DNT exposure, determined genotoxic effects of 24DNT and hypothesized its mechanism of action at the pathway level.

Work in this study involves pathway-based network analysis using DEGs in liver tissue of animals exposed to 3 different concentrations of 2,4DNT. All the DEGs are visualized as a pathway - protein frequency count matrix (PPFCM). The PPFCM generated using these DEGs upon 24DNT treatment shows the top four pathways induced: (a) long term depression; (b) breast cancer regulation by stathmin1; (c) retinol metabolism; and (d) WNT Signaling. The PI3K pathway is an intricate signaling cascade that regulates cell survival and growth under normal, as well as pathological conditions. In fact, the PI3K pathway is mutated in more cancer patients than any other [18]. The signaling network is balanced by the PTEN tumor suppressor protein. PTEN (Phosphatase and Tensin Homologue Deleted from Chromosome-10) is recognized as one of the most frequently mutated tumor suppressors in human cancer and has also been associated with neurological diseases like autism [19].

Figure 1 contains important pathways responsible for maintaining essential functions of normal cells. GSK3 and WNT signaling pathways are known for their roles in embryogenesis and cancer, but they are also involved in normal physiological processes in adult animals. GSK3 is a key regulator in several physiological processes such as cell cycle, oncogenesis and apoptosis in neuronal cells and VSMC (Vascular Smooth Muscle Cells) during hypoxia [20]. GSK-3b (a splice variant) plays a key inhibitory role in the Wnt signaling pathway. Wnt genes encode a large family of secreted, cysteine-rich proteins that are important in development and in maintenance of adult tissues. Abnormalities in Wnt signaling are reported to promote both human degenerative diseases and cancer [21].

Abnormalities in pathways that use GSK-3 as a regulator have been linked to several disease conditions, particularly in non-insulin-dependent diabetes mellitus, Alzheimer’s disease, developmental disorders, and cancer [22]. A decrease in both GSK3 and WNT signaling pathways may therefore be a mechanism contributing to carcinogenesis upon 2,4DNT insult. The above observations validate the existing knowledge and provide new leads to discover the action of 2,4DNT. Pathways such as these also hint that primary targets of 2,4DNT’s toxicity are the hematopoietic, cardiovascular, nervous and reproductive system.

A pathway merging diagram has been developed using the top 20 pathways from the pathway - protein frequency count matrix (PPFCM). This causal network shows concordance with pathways affected or diseases caused by 2,4DNT exposure and gives clues about other diseases. The network involves pathways such as ERK, PI3K and AKT signaling known to be involved in leukemia and bladder cancer which are also diseases associated with the cytotoxic effects of 2,4DNT. The presence of other pathways such as ‘calcium signaling pathway’ and ‘retinol metabolism’ may indicate additional diseases caused due to the mutagenic effects of 2,4DNT. This network may therefore warrant further investigation to discover novel effects of 2,4DNT.

Our reported observations strengthen the necessity of integrating pathways from heterogeneous sources and also validate the existing literature knowledge. HPD pathways in this study provide a good meta-model that connects our fragmented pathway knowledge together in pathway merging networks. A global perspective, supported by integration of otherwise incompatible pathways from different sources, enhances the chance of exposing novel insights in the search for disease drug targets and biomarkers. Our results provide clues about the mechanism action of energetic compounds such as 2,4DNT and explain their genotoxic effects at the pathway level.

These findings strongly support the potential of this framework for evaluating functional genomics data using pathway networks. In addition to validating existing literature findings; new knowledge can be derived by adopting the methods used in our case study.

## Material and methods

### Chemicals

2,4DNT (97%) was purchased from Sigma-Aldrich (St. Louis, MO).

### Animals and treatment

Female Sprague-Dawley rats (175-225 grams) were from the in- house breeding colony (College of Pharmacy, University of Louisiana at Monroe [ULM] and treated in accordance with the *Guide for Use and Care of Animals*[23]. Breeders were from Harlan-Sprague Dawley in Madison, WI. Housing consisted of a 12 h light/dark cycle with *ad libitum* access to tap water and rodent chow (Harlan/Teklad 7012, Madison, WI). Rats were housed individually in polycarbonate cages on hardwood bedding (Sani-chips, Harlan/Tekland, Madison, WI) one week prior to treatment. Food was withdrawn the night before treatments [vehicle control (5% DMSO in corn oil), or one of four doses (4.98, 49.8, 99.5, or 199 mg/kg) of 2,4DNT in 5% DMSO in corn oil] which were administered by gavage between 8 and 10 AM. Study protocols were preapproved by the ULM Animal Care and Use Committee.

### Microarray experimental design

Changes in gene expression were tested using Agilent commercial whole rat genome microarrays (4 X 44K). In this project, four doses plus a vehicle control were employed for 2,4-DNT at 24h. The dose selection was based on the LD 50 data for each compound. Except controls, the lowest dose, the second lowest dose, the second highest dose and the highest dose were selected. Four biological replicates of this design were conducted, each using different animals.

### Total RNA extraction

Total RNA was extracted from about 30mg of liver tissue. Tissues were homogenized in the lysis buffer with FAST Prep-24 from MP before using RNeasy kits (Qiagen). Total RNA concentrations were measured using a NanoDrop® ND-1000 Spectrophotometer (NanoDrop Technologies, Wilmington, DE, USA). The integrity and quality of total RNA was checked on an Agilent 2100 Bioanalyzer (Palo Alto, CA). The gel-like images generated by the Bioanalyzer show that total RNAs have two bands, representing the 18S and 26S RNA of mammalian RNA . Nuclease-free water (Ambion) was used to elute total RNA.

### Microarray hybridization

Rat whole genome oligo arrays in the format of 4X44K were purchased from Agilent Technologies. Sample cRNA synthesis, labeling, hybridization and microarray processing were performed according to manufacturer’s protocol "One-Color Microarray-Based Gene Expression Analysis" (version 1.0). The labeling reactions were performed using the Agilent Low RNA Input Linear Amplification Kit in the presence of cyanine 3-CTP. The labeled cRNA from each labeling reaction was hybridized to individual arrays at 65 °C for 17 hours using Agilent’s Gene Expression Hybridization Kit. After washing, the arrays were scanned using a GenePix 4200AL scanner (Molecular Device Inc., Sunnyvale, CA). The Feature extraction software (V. 9.5.1) from Agilent was used to automatically find and place microarray grids, reject outlier pixels, accurately determine feature intensities and ratios, flag outlier pixels, and calculate statistical confidences.

### Microarray data pre-processing

Microarray data analyses were processed with GeneSpring version 7.0 and 10.0. The sample quality control was based on the Pearson correlation of a sample with other samples in the whole experiment. If the average Pearson correlation with other samples was less than 80%, the sample was excluded for further analysis. If the scanned intensity was less than 5.0 for a probe, it was transformed to 5. A perchip (within) array normalization was performed using 50 percentile values of all the probe values in the array. Per gene (between) array normalization was also applied using the median value of a gene across all samples in the experiment. Probe features were first filtered using flags. A "present" or "absent" flag was defined using the Agilent *Feature Extraction 9.5.1* software. Only a probe that had present flags in at least 50% samples of all the arrays was kept for further analyses. Data were subsequently log (base 2) transformed for statistical analyses.

### Rat Microarray Analysis

We use genome-scale gene expression data of liver cells from rats treated with 4.98, 49.8, 99.5, or 199 mg/kg 2,4DNT to perform pathway analysis using human pathway database (HPD) (Chowbina, Wu et al. 2009), build dose-specific protein-protein association matrix and dose- response networks. Therefore, the driving motivation is that knowledge of these pathways / protein-protein interaction networks will help clarify and interpret physiological response**s** to 2,4DNT, which will advance our understanding of the health consequences of 2,4DNT treatment.

### Significant Analysis of Microarray (SAM)

Significance analysis of microarrays SAM software [24] was used in two-class mode to determine the list of genes best able to distinguish genes in control and each of the three 2,4DNT dose groups which were run on separate Microsoft Excel spreadsheets. The log (base 2) normalized gene expression data of rat liver was imported into a SAM plug-in of Microsoft Excel. The SAM method (Tusher, Tibshirani et al. 2001), uses a modified t-test statistic, with sample-label permutations to evaluate statistical significance. Delta was chosen to limit the output gene list so that fewer than 5% predicted false-positives are included. Significant positively and negatively correlated genes whose mean expression in the 2,4DNT group is greater or lesser than those in the CONTROL group with a fold change of at least 2 and q-value less than 0.01 are selected for further analysis. Differentially expressed genes for each dose are uploaded into HPD [17] and these genes are visualized as a pathway – protein frequency count matrix (PPFCM) using the TIGR MeV (MultiExperiment Viewer) [25].

## Competing interests

The authors declare that they have no competing interests.

## Authors’ contributions

JS participated in the method development, pathway analysis and manuscript drafting. YD designed the project, carried out the data analysis, and wrote part of the manuscript. JA, XW participated in data analysis and processing. XG and BLE conducted microarray experiment. MSW and SAM performed animal treatment. EJP coordinated and directed the overall project. JYC initiated the project and performed direct algorithm development, as well as contributed to the writing of the manuscript. All authors read and approved the final manuscript.
